# ctDNA responds to neoadjuvant treatment in locally advanced rectal cancer

**DOI:** 10.1007/s00432-024-05944-7

**Published:** 2024-09-22

**Authors:** Florian Bürtin, Liema Elias, Sebastian Hinz, Michael Forster, Guido Hildebrandt, Bernd Frerker, Felix Bock

**Affiliations:** 1 Department of General Surgery, Rostock University Medical Center, Rostock, Germany; 2 Institute of Clinical Molecular Biology, Christian-Albrechts-University and University Medical Center Schleswig- Holstein, Kiel, Germany; 3 Department of Radiotherapy and Radiation Oncology, Rostock University Medical Center, Südring 75, 18059 Rostock, Germany

**Keywords:** Locally advanced rectal cancer, Neoadjuvant therapy, Radiotherapy, ctDNA, Liquid biopsy

## Abstract

**Background:**

Liquid biopsy is a minimally invasive procedure investigating tumor mutations.

**Methods:**

In our retrospective study, we investigated whether molecular therapy monitoring of patients receiving neoadjuvant radio(chemo)therapy on a daily routine is possible in 17 patients with locally advanced rectal cancer. Six patients received short-course radiotherapy (5 × 5 Gy) with subsequent surgery, six patients were treated according RAPIDO protocol with short-course radiotherapy followed by chemotherapy (FOLFOX4) and subsequent surgery and five patients received conventional neoadjuvant radiochemotherapy with 5-FU followed by surgery. Response was assessed by Dworak. Liquid biopsies were taken before and immediately after neoadjuvant radiotherapy to isolate and ultradeeply sequence cell free DNA with a panel of 127 genes. Somatic mutations were determined bioinformatically by comparison with normal DNA from leukocytes to distinguish them from germline variants or aging mutations.

**Results:**

In 12 patients (71%) at least one somatic mutation was detected. In 8/12 patients a decrease and in 4/12 an increase or mixed response in ctDNA was seen. Statistical correlation between ctDNA analysis and clinical response could not be seen.

**Conclusion:**

ctDNA is responding to neoadjuvant therapy and liquid biopsy is easily integrated into a daily routine. As part of translational research this protocol leaves room for further investigations.

## Introduction

Colorectal carcinoma (CRC) is currently the second most common cancer in women and the third most common in men and accounts for approximately 8% of all cancer-related deaths in Germany (Rki 2019). Approximately a quarter of affected women and about a third of affected men have tumors localized in the rectum (Arndt et al. 2021). It is worth noting that while only a minority of patients develop rectal cancer before the age of 50, there has been a significant increase in the incidence among individuals aged 20–50 years in recent years (Emrich and Kraywinkel 2020). The current 5-year survival rates for UICC stages I to IV in Germany are 94%, 86%, 72%, and 16% for women and 92%, 82%, 73%, and 18% for men, respectively (Rki 2019). Rectal carcinomas infiltrating the mesorectum or beyond (UICC stage II) and with lymph node involvement are referred to as locally advanced rectal carcinoma (LARC).

In recent decades, therapy for rectal carcinoma has undergone continuous evolution with a steady improvement in disease-free survival. While the introduction of total mesorectal excision (TME) certainly represented a significant milestone in surgical therapy by already significantly reducing local recurrence rates, the implementation of neoadjuvant therapy for LARC using short-course radiation (SCRT) or fractionated radiochemotherapy (fRCT) has led to even further improvements in terms of local recurrences and additionally improved rates of sphincter preserving resections (Bujko et al. 2006; Oechsle et al. 2009; Maurer et al. 2011). Since 12–15% of patients treated with fRCT achieve pathological complete response (pCR), defined as a complete lack of vital tumor cells in the resected TME specimen, the watch-and-wait-strategy emerged as a treatment alternative for patients with clinical complete response (cCR), assessed by endoscopy and sectional imaging, omitting TME after fRCT (Habr-Gama et al. 2004). Since disease-free survival of locally advanced rectal cancer (LARC) patients is largely determined by the occurrence of metastases rather than local recurrence, the total neoadjuvant therapy (TNT) aims to intensify tumor and possible micrometastases exposure to chemotherapy (Fokas et al. 2014). The international RAPIDO trial compared TNT with 5 × 5 Gy radiotherapy followed by chemotherapy with 5FU, leucovorin and oxaliplatin (FOLFOX4), followed by surgery, against the fRCT arm (2021). The French PRODIGE23 study on the other hand, compared total TNT using induction chemotherapy (a quadruple combination of 5FU, leucovorin, irinotecan, and oxaliplatin) followed by fRCT, subsequent surgery, and adjuvant chemotherapy to fRCT followed by surgery and intensified adjuvant chemotherapy. Once again, TNT proved superior in terms of disease-free survival, pCR and overall survival rates (Conroy et al. 2023). The RAPIDO trial, nevertheless, unfortunately showed higher local recurrence rates within the TNT arm in the five year follow-up (Dijkstra et al. 2023).

On the one hand, the rate of non-responders ranges from 11 to 14% for TNT, as observed in the CAO/ARO/AIO-12 trial, and up to 54% for fCRT (Battersby et al. 2018; Fokas et al. 2022). On the other hand, despite extensive diagnostics, there remains ambiguity between cCR and pCR. While 25% of patients achieving cCR experience local recurrence, up to 15% of patients with cPR undergo surgery due to false-positive restaging (Maas et al. 2015; van der Valk et al. 2018). Consequently, a significant portion of rectal cancer patients undergoes considerable toxicity without benefiting from it, while another group undergoes rectal resection even though organ preservation could potentially be accomplished. Therefore, further methods are needed to predict treatment response and failure, respectively, increase the diagnostic accuracy between cCR and pCR, and enable early detection of post-therapeutic recurrences.

The term liquid biopsy encompasses various minimally invasive procedures for the detection of disease-specific biomarkers in cancer patients’ bodily fluids, primarily in the blood. This involves the detection, characterization, and quantification of circulating tumor cells (CTCs), peptides, microRNAs (miRNAs), cell-free DNA (cfDNA) or circulating tumor DNA (ctDNA) (Fernández-Lázaro et al. 2020). While traditional biopsy involves a spatially limited tumor area and is hardly repeatable during ongoing therapy, ctDNA enables the capture of molecular heterogeneity, clonal selection, and secondary resistances (Bettegowda et al. 2014). The aim of our study was to evaluate the applicability of ctDNA detection and quantification in the prediction of treatment response in a heterogeneous, real-life patient population undergoing neoadjuvant treatment for LARC.

## Materials and methods

### Study design

Blood samples were collected in the routine clinical workflow at the University Medical Centre Rostock. All patients gave written informed consent. The study was conducted in accordance with the Declaration of Helsinki and approved by the Institutional Review Board of the University Medical Center Rostock (A2020–0193).

This study is a prospective, real-world, single-center analysis including 17 patients with histologically confirmed locally advanced non-metastatic rectal adenocarcinoma with the necessity of a neoadjuvant radio(chemo)therapy prior to surgery.

Following physicians’ choice, patients were treated with SCRT consisting of 5 × 5 Gy followed by immediate surgery, with SCRT followed by FOLFOX4 with subsequent surgery representing a total neoadjuvant approach according to the RAPIDO trial or with neoadjuvant fRCT (50.4 Gy in 28 fractions with concomitant 5FU) followed by surgery.

Blood samples were collected in Streck cfDNA BCT tubes (Streck, Inc.) in duplicates, i.e. two times 9 ml blood per sampling time point. The first sample duplicates were obtained before the first radiotherapy fraction and the second sample duplicates were obtained within an hour after the end of the final radiotherapy fraction. The samples were pseudonymized by numbers and the tubes were tracked into the research database laboratory information system (LIMS) with the help of barcode stickers. The tubes were sent to the Institute of Clinical Molecular Biology in Kiel for further processing according to the manufacturer’s instructions, as previously described (Richter et al. 2023). In brief, the blood was separated into three layers by centrifugation: Erythrocytes, leukocytes (buffycoat) and plasma. The leukocytes and the plasma were each stored frozen at -20 °C until further processing. DNA was isolated from the blood plasma and buffycoat using two specific kits and NGS libraries were prepared from the DNA using two specific protocols. Hybridization-based targeted capture of the libraries was performed with the IDT xGen Pan-Cancer panel v1.5 to focus on 127 cancer genes identified by The Cancer Genome Atlas (TCGA), totaling in a target region size of 800 kilobases. Sequencing was performed on Illumina NovaSeq 6000. Sequences were aligned to hg19 using bwa mem (Li and Durbin 2009) and multi-library data analysis of the cfDNA library duplicates at two time points versus buffycoat DNA libraries was performed with GenSearchNGS (Phenosystems S.A.) as previously described (Richter et al. 2023). Cell-free DNA sequences from the plasma were analyzed to search for tumor components, i.e. somatic mutations that were not present in the leukocyte DNA. However, recurrent technical artefacts are known to occur in sequencing studies. Therefore, an additional filtering technique was performed to identify potential false positives in the list of obtained somatic mutations: The variant allele frequency (VAF) of each somatic mutation detected in cfDNA was compared to the number of reference and alt reads at the corresponding genomic position in all buffycoat DNA libraries, using the GenSearchNGS Variant Verifier tool. The somatic variant was deemed true if its VAF was greater than two times the 95%-percentile-VAF of the buffycoat libraries, as computed with a python script (https://github.com/ikmb/cfDNA-quantile). The filtered mutations were graphically summarized into a genomic landscape plot (https://fluxus.pythonanywhere.com/plot/genomic_landscape) and their temporal changes between the two time points were summarized in a stacked-bar-plot (https://fluxus.pythonanywhere.com/plot/stacked_bar). The statistical analysis of the correlations between two factors was conducted by calculating the two tailed p-value using Fisher’s exact test using Graphpad Prism.

### Detection of circulating cell-free tumor DNA

The presence of cell-free tumor DNA was inferred bioinformatically by somatic mutation detection in sequences from cfDNA sample duplicates versus sequences from leukocytes from the same patient.

The technical limit of detection is generally worse than the biological limit, because PCR or sequencing errors obscure the biological signal (Frank et al. 2021). Therefore, we performed a multi-step analysis of the sequence data to first find the potential somatic mutations, and then to validate them in two stages.

Following the isolation and sequencing of cfDNA, we aligned the sequence data and then conducted a comparison between the cfDNA isolated from samples obtained before and after neoadjuvant therapy with normal DNA (leukocyte-DNA). Using GenSearchNGS software, we identified circulating tumor DNA (ctDNA) within the cfDNA. On a 10-core server, the software needed approx. 8–12 h to generate the mutation list for the total of 17 patients, after which the list could be filtered and annotated. In addition, the online annotation of the filtered variants for each patient took approx. 10–20 min.

## Results

### Consort diagram

**Fig. 1:**
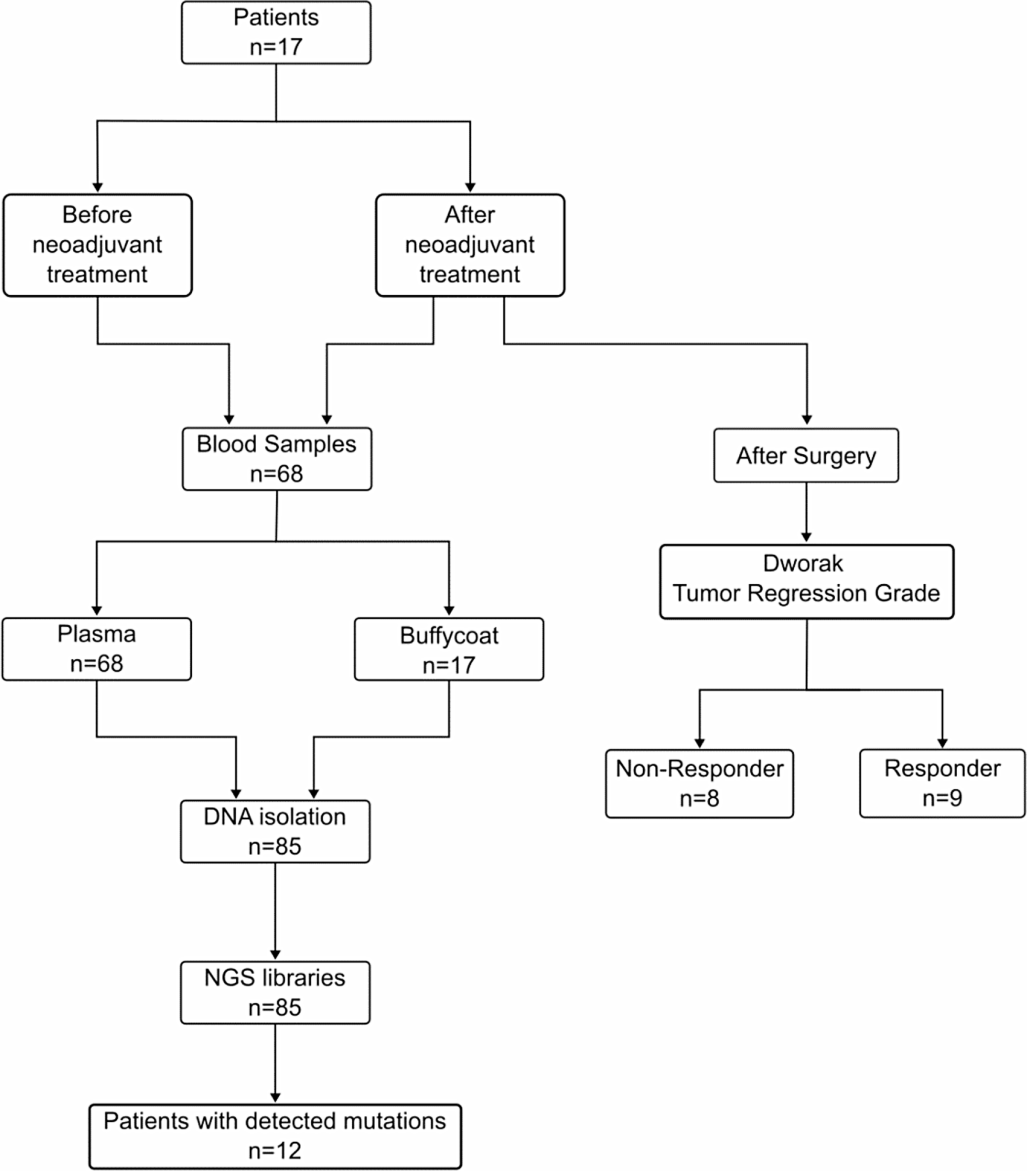
Consort diagram of CRC patient samples and workflow. Blood samples were taken in duplicates. Plasma was used from each duplicate for mutation detection in cell free DNA. Buffycoat was used from one time point to provide the background of germline variants and to filter for variants of clonal haematopoesis of indeterminate potential (e.g. aging-related mutations in leukocyte subfractions). Next Generation Sequencing (NGS) libraries were sequenced and jointly analyzed to detect somatic mutations

The study workflow and resulting number of samples, sequencing libraries and patients with mutations is shown in the consort diagram in Fig. 1.

### Study population

Median age at the time of diagnosis was 65 years (range: 56–97). 5/17 patients were treated with SCRT followed by immediate surgery, 6/17 with SCRT followed by FOLFOX4 with subsequent surgery according to the RAPIDO trial and 6/17 with neoadjuvant fRCT followed by surgery. The average distance from the distal tumor margin to the anocutaneous line was 6 ± 3,25 cm. While 11 patients underwent low anterior rectal resection, abdominoperineal resection was necessary in 6 cases. Response rates were evaluated according to Dworak and patients were divided into a non-responder (*n* = 8) and a responder group (*n* = 9). Table 1 shows patients and treatment characteristics.

**Table 1:** Patient demographics and clinical characteristics of the study population of CRC patients

		*N* (%)
All		17 (100)
Sex	Male	12 (70.6)
	Female	5 (29.4)
Age [years]	< 65	8 (47.1)
	> 65	9 (52.9)
cT category	cT2	0 (n.a.)
	cT3	8 (47.1)
	cT4	9 (52.9)
ypT category	ypT2	1 (5.9)
	ypT3	8 (47.05)
	ypT4	8 (47.05)
cN category	cN0	0 (n.a.)
	cN1	10 (58.8)
	cN2	7 (41.2)
ypN category	ypN0	3 (17.6)
	ypN1	9 (52.9)
	ypN2	5 (29.4)
p/cM category	cM0	15 (88.2)
	pM1	2 (11.8)
pUICC-Stage	II	8 (47.05)
	III IV	8 (47.05) 1 (5.9)
Neoadjuvant treatment	RAPIDO fRCT SCRT	6 (35.3) 6 (35.3) 5 (29.4)
Response Dworak	Yes No	9 (52.9) 8 (47.05)
Downstaging	Yes No	13 (76.5) 2 (23.5)

### Dworak tumor regression grade and downstaging

After neoadjuvant therapy, all 17 patients underwent surgery. An experienced pathologist, who was blinded to the clinical results and cell-free DNA information, comprehensively evaluated each postoperative specimen. Tumor regression grade (TRG) was assessed by histologic evaluation of the surgical specimens according to the guidelines described by Dworak et al. (Dworak et al. 1997) with responders having TRG 3 or 4, and non-responders having TRG 1, as listed in Table 2. Furthermore, we documented clinical downstaging, which occurred when there was a decrease in both T and N staging in our study cohort. Based on our analysis of the tumor staging data, 13 out of 17 patients showed clinical downstaging, whereas the remaining 4 patients did not show this phenomenon (Table 2).

**Table 2:** Patient demographics and clinical characteristics of the study population of CRC patients

Patient ID	Protocol	Dworak (TRG)	Downstaging
HROC520 HROC528 HROC535 HROC539 HROC540 HROC547 HROC554 HROC556 HROC566	fRCT RAPIDO RAPIDO fRCT SCRT RAPIDO fRCT fRCT RAPIDO	4 3 3 4 3 4 4 3 3	Yes Yes Yes Yes Yes Yes Yes Yes Yes
HROC534 HROC536 HROC544 HROC558 HROC562 HROC574 HROC586 HROC561	fRCT SCRT RAPIDO SCRT RAPIDO SCRT SCRT SCRT	1 1 1 1 1 1 1 1	No Yes No No Yes No Yes Yes

### cfDNA detection and -changes

The amount of cfDNA extracted from plasma in a 10 ml blood collection tube is summarized in Table 3. Before neoadjuvant therapy (t0), it ranged from 6.4 to 176 ng (median 29 ng) and in the sample taken right after the last radiation therapy (t1) it ranged from 5.25 to 366 ng (median 44 ng). The expected number of diploid genome copies in each plasma sample ranged from 983 to 27,035 copies at t0 and from 806 to 56,221 copies at t1 (Supplemental Table 3), based on the average female genome weight of 6.51 pg (Piovesan et al. 2019). The biological tumor detection limit for the plasma samples with the lowest cfDNA amounts are therefore: 1 haploid tumor genome in 1966 haploid genomes (VAF 0.05%) for 586-t0 with 6.4 ng cfDNA, and 1 haploid tumor genome in 1612 haploid genome copies (VAF 0.06%) for 566-t1 with 5.25 ng cfDNA.

**Table 3:** Measured cfDNA before (t0) and after (t1) neoadjuvant radiotherapy

Patient ID	cfDNA-t0 (ng)	cfDNA-t1 (ng)
HROC520	46.8	41.4
HROC528	16.4	53.1
HROC534	21.9	35.3
HROC 535	53.4	32.0
HROC 536	19.6	53.2
HROC539	19.6	49.8
HROC540	44.7	69.3
HROC544	34.5	366.0
HROC547	10.2	43.9
HROC554	56.7	21.2
HROC556	39.2	58.3
HROC558	45.5	48.2
HROC562	176.0	137.9
HROC566	18.6	5.3
HROC574	18.1	21.2
HROC586	6.4	19.9
HROC601	29.2	39.2

**Fig. 2:**
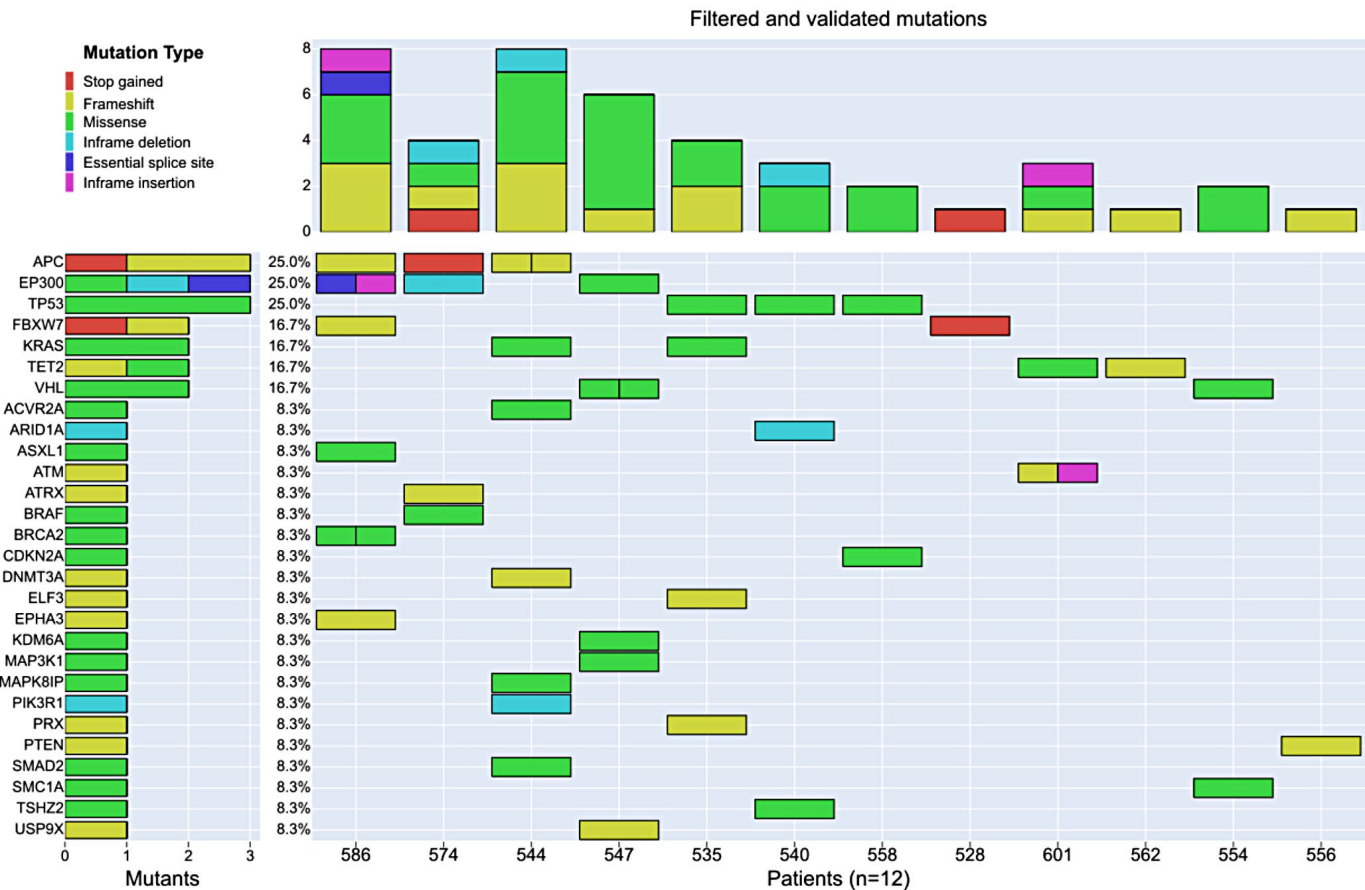
Oncoplot of detected somatic mutations and copy number changes in 17 LARC patients. The most frequent mutation type was a missense mutation (*n* = 22), followed by frameshift mutations (*n* = 13), Inframe deletion or insertion (5), stop gained mutations (*n* = 2) and Essential splice site (*n* = 1)

We observed an average of 14 potential somatic mutations per patient. However, through the subsequent two-stage validation process, only an average of 2 mutations per patient were ultimately confirmed as validated mutations. For 12 patients (71%) at least one somatic mutation was detected. In contrast no confirmed mutation was found in 5 patients (29%). We detected a total of 28 different confirmed mutations. The most frequently mutated genes were *APC*, *EP300*, *TET2*, and *TP53*, each with a frequency of 25%, followed by *FBXW7*, *KRAS*, and *VHL* with a frequency of 16.7% (Fig. 2). All other mutations occurred with an equal frequency of 8.3%.

### VAF-changes

**Fig. 3:**
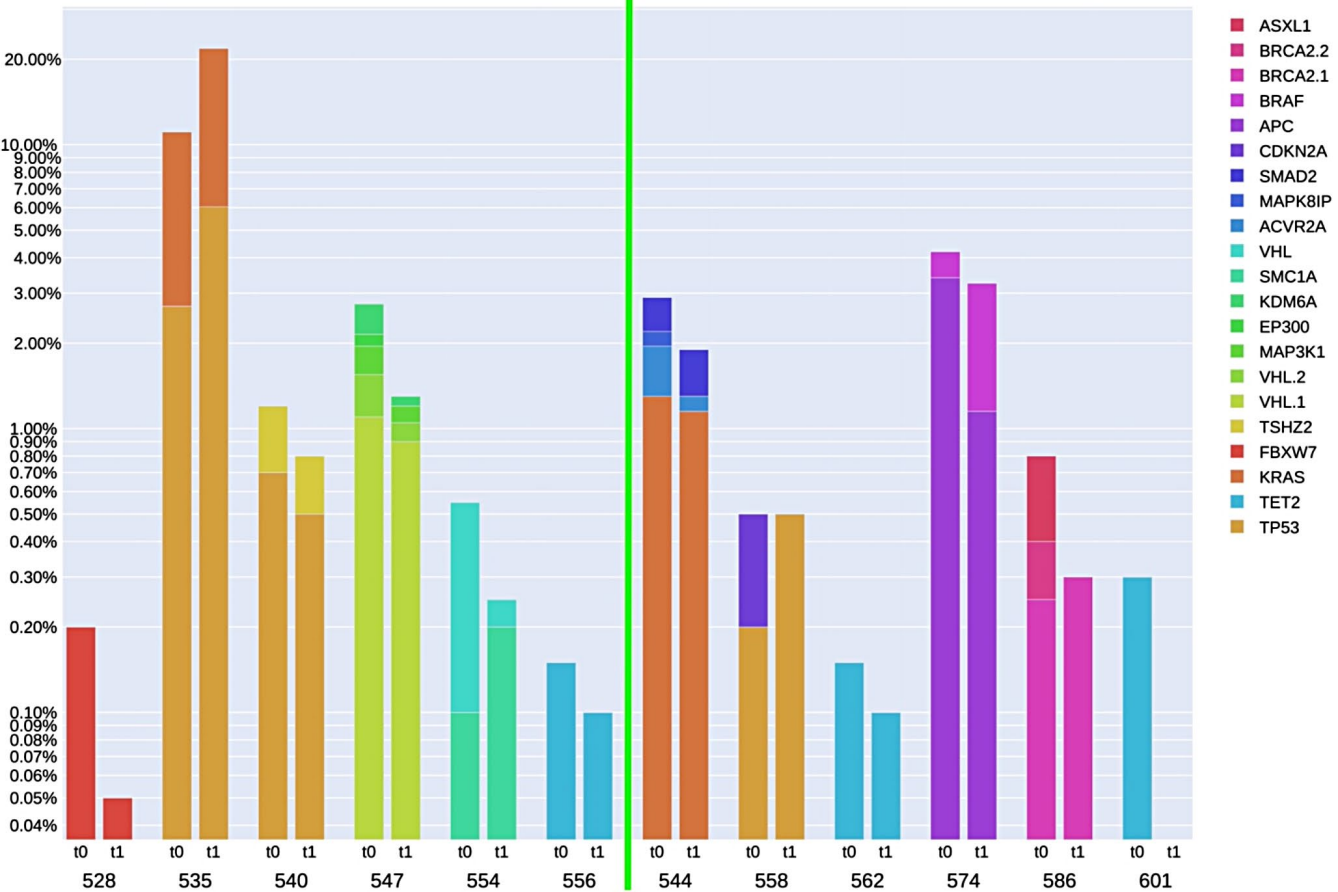
Detected ctDNA before neoadjuvant treatment (t0) and after final radiation (t1). The figure shows genes in which mutations were detected in the cfDNA and the VAFs of the mutations. Left half: Dworak-responders; right half: Dworak-non-responders

Subsequently, we analyzed the change in variant allele frequency (VAF) between t0 before neoadjuvant treatment and t1 after the final neoadjuvant radiation therapy. At t0 the somatic mutation VAFs ranged from 0.1 to 8.4% (median 0.4%) and at t1 they ranged from 0.0 to 15.8% (median 0.15%). In 8 out of 12 patients the ctDNA decreased, in the other 4 patients mutation-specific increase as well as decrease in ctDNA was seen (Fig. 3). No statistically significant association was found between Tumor Regression Grade according to Dworak and the change in ctDNA in the blood plasma between t0 and t1.

## Discussion

With the increasing implementation of TNT, there is an urgent need for precise diagnostic tools for the early detection of non-responders, thereby sparing unnecessary toxicity and the delay of surgical treatment for those who do not benefit from TNT. Furthermore, a non-invasive method for monitoring the course in patients with cCR would be desirable to facilitate the treatment decision between a watch-and-wait-strategy and surgery.

Previous studies focusing on primarily resected localized colorectal cancer have already shown benefits in ctDNA-guided management regarding the detection of minimal residual disease and, therefore, being essential for selecting patients requiring further treatments (Peach et al. 2010; Iinuma et al. 2011; Tie et al. 2016, 2021; Reinert et al. 2019; Tarazona et al. 2019; Dasari et al. 2020; Parikh et al. 2021; Benhaim et al. 2021; Kotani et al. 2023a). Despite this cumulating data in favor of ctDNA monitoring in the adjuvant setting, there remains a lack of evidence in the neoadjuvant approach. Therefore, we evaluated the applicability of ctDNA detection and quantification in patients undergoing neoadjuvante treatment for LARC.

The overall detection rate of ctDNA in our small pilot study was approximately 70%, which is comparable to previous publications (Murahashi et al. 2020a; Zhou et al. 2021; Kotani et al. 2023b). In a relatively large NGS-based study, Tie et al. were able to detect ctDNA in 77% of LARC patients. Changes in ctDNA levels after neoadjuvant therapy were not associated with pCR-rates, however, postoperative detection of ctDNA showed a high predictive value for tumor recurrence (Tie et al. 2019). After sequencing tumor samples from 47 rectal cancer patients, a British research group detected the mutation with the highest variant allele frequency in plasma using ddPCR in 74% of patients before neoadjuvant chemoradiotherapy (nRCT) (Khakoo et al. 2020). Both the detection of ctDNA after completion of nRCT and after surgery were associated with significantly shorter metastasis-free survival. However, in patients who opted for a watch-and-wait approach, the detection of local recurrences using ctDNA was not successful. Conversely, Zhou et al. (Zhou et al. 2021) were able to detect ctDNA in 75% of rectal cancer patients before nRCT using targeted capture sequencing of 1021 genes. While the detection of ctDNA before the commencement of nRCT showed no correlation with treatment response, ctDNA detection rates were significantly lower in preoperative samples of patients achieving pathological complete and good response (Zhou et al. 2021). Likewise, in a Chinese study analyzing ctDNA changes during nRCT of LARC, the baseline concentration of ctDNA did not influence therapy response. However, the decrease of ctDNA below the detection limit was associated with a complete pathological response whereas an increase in acquired mutations correlated with worse tumor regression grades (Wang et al. 2021). In contrast to the study by Zhou et al. (Zhou et al. 2021), where all patients without detectable ctDNA belonged to the group of complete responders, our patient cohort did not show any association between ctDNA detection and response (ctDNA-negative: 3 responder vs. 2 non-responder).

We observed a decrease in VAF in 8 out of 12 patients, an increase in one patient and a combination of both increase and decrease in 3 patients. No association between treatment response and changes in VAF could be found. Although a decrease of VAF can be attributed to treatment-associated tumor shrinkage, it is not necessarily associated with the grade of pathological response. Despite a significant reduction between baseline and preoperative VAF in nRCT treated LARC patients achieving CR being noticed by Murahashi et al., a large proportion of non-responders showed a similar decrease in VAF (Murahashi et al. 2020b). In our study, somatic mutations were detected in *APC*, *EP300*, *TET2*, and *TP53*, each with a frequency of 25%, followed by *FBXW7*, *KRAS*, and *VHL* with a frequency of 16.7%. All other mutations occurred with an equal frequency of 8.3%. While *APC*, *TP53*, *KRAS* and *FBXW7* are commonly mutated genes found in CRC, the relatively high frequency of *EP300* and *TET2* mutations is somewhat surprising (Muzny et al. 2012a). *EP300* codes a histone acetyltransferase involved in gene expression regulation by acting as a transcriptional coactivator and frequently expressed in CRC (Ishihama et al. 2007). However, the frequency and role of somatic mutations of *EP300* in colorectal cancer is not fully elucidated, but an association with microsatellite instability (MSI) is described in the literature (Kim et al. 2013). *TET2* mutations are frequent driver mutations in myeloid malignancies, but rather uncommon in CRC, in the latter also exclusively in hypermutated tumors (Muzny et al. 2012b; Ferrone et al. 2020). Since the VAF profiles of the respective patients do not indicate any evidence of hypermutation, an MSI-associated *EP300* mutation appears unlikely. An increase or nearly constant VAF of *TP53* mutations was observed in 3 patients, suggesting a positive selection pressure on *TP53*-mutated tumors after neoadjuvant treatment (Sakai et al. 2014). However, contrary to previous findings, neither *TP53* mutations alone, nor the co-mutation of *TP53* and *KRAS* were associated with treatment resistance in our cohort (Kamran et al. 2019; Sclafani et al. 2020).

In summary, our study has several limitations. Primarily, due to the small group size, it lacks statistical power. Additionally, another measurement point postoperatively to evaluate ctDNA as a predictive parameter for DFS would have been beneficial. Both circumstances are due to the pronounced cost intensity of the methodology as well as the considerable logistical effort. Moreover, a comparison of the mutational profiles between the blood samples and the original tumor tissue could have strengthened the validity of our data; however, it was not possible due to decentralized pathological processing of the surgical specimens. Nevertheless, we were able to demonstrate that the methodology of unbiased NGS analysis is applicable to a small real-life patient population and exhibits relevant sensitivity. Although ctDNA analysis is currently not suitable as a gatekeeper for neoadjuvant therapy or the watch-and-wait concept, it may still represent a valuable addition to traditional tumor markers and imaging techniques in the context of follow-up care.

## Conclusions

Circulating tumor DNA is responding to neoadjuvant therapy and liquid biopsy is easily integrated into a daily routine. Nevertheless, the lack of validated and standardized protocols for ctDNA detection limits the comparability of individual studies. Conclusively, this study underlines the possible opportunities and challenges of ctDNA in monitoring the neoadjuvant setting in locally advanced rectal cancer. As part of translational research this protocol leaves room for further investigations correlating ctDNA changes und mutations with oncologic outcomes in a higher number of patients.

## Data Availability

No datasets were generated or analysed during the current study.
